# Surveillance of *Legionella pneumophila*: Detection in Public Swimming Pool Environment

**DOI:** 10.3390/microorganisms10122429

**Published:** 2022-12-08

**Authors:** Darija Vukić Lušić, Vanda Piškur, Arijana Cenov, Dijana Tomić Linšak, Dalibor Broznić, Marin Glad, Željko Linšak

**Affiliations:** 1 Department of Environmental Health, Faculty of Medicine, University of Rijeka, Brace Branchetta 20, 51000 Rijeka, Croatia; 2 Department of Environmental Health, Teaching Institute of Public Health of Primorje-Gorski Kotar County, Krešimirova 52a, 51000 Rijeka, Croatia; 3 Department for Medical Chemistry, Biochemistry and Clinical Chemistry, Faculty of Medicine, University of Rijeka, Brace Branchetta 20, 51000 Rijeka, Croatia

**Keywords:** *Legionella* spp., physicochemical parameters, prevention, recreational water, swimming pool water

## Abstract

The bacterium *Legionella pneumophila* is a ubiquitous microorganism naturally present in water environments. The actual presence of this opportunistic premise plumbing pathogen in recreational swimming pools and hot tubs in the northwestern part of Croatia has not been investigated. This study aimed to analyze the presence of the opportunistic pathogen *L. pneumophila* in public swimming pool water in Primorje-Gorski Kotar County (N = 4587) over a four-year period (2018–2021). Additionally, the second aim was to investigate the connection between the presence of *L. pneumophila* and pool water physicochemical parameters using mathematical predictive models. The presence of *L. pneumophila* was detected in six pool samples. Five positive samples were found in the water of indoor hot tubs filled with fresh water, and one positive sample in an outdoor recreational saltwater pool. A predictive mathematical model showed the simultaneous influence of chemical parameters dominated by the temperature in saltwater and freshwater pools, as well as the significant influence of free residual chlorine and trihalomethanes. Our results pointed out that keeping all physicochemical parameters in perfect harmony is necessary to reach the best disinfection procedure and to avoid the optimum conditions for *L. pneumophila* occurrence.

## 1. Introduction

One of the most common opportunistic premise plumbing pathogens (OPPPs) that often remain present in water supply systems is a Gram-negative bacteria belonging to the family *Legionellacea*, which can cause severe health problems known as the disease Legionellosis [1]. Most cases mentioned in the published literature correlate to *Legionella pneumophila* serogroup 1, but other species and serogroups are also associated with human diseases, such as *L. dumoffii*, *L. micdadei*, and *L. longbeachae* [2]. Up to 8% of Legionnaires’ disease (LD) cases in European Union countries and the European Economic Area (EU/EAA) end fatally, according to the surveillance report by the European Centre for Disease Prevention and Control (ECDC) [3]. Legionellosis is an infection, with symptoms ranging from mild febrile illness (Pontiac fever) to severe pneumonia, with potentially fatal outcomes (Legionnaires’ disease) [4].

This airborne microorganism is transmitted by the inhalation of contaminated aerosols produced by human-made water systems such as residential water supply facilities, cooling towers, spa pools, fountains, and misting devices [5,6]. Moreover, some of the most important characteristics of OPPPs is their tendency to exist either as free-floating cells or enclosed within an architectural structure known as biofilm [7] and, of course, their thermotolerance and viability between 25 and 37 °C, the preferable and most usual temperature range in swimming pools, hot tubs, and similar aquatic environments. *Legionella* biofilm formation is influenced by hydrodynamic conditions, microflora, sediment, temperature, and physicochemical properties of the water [8]. Disinfection is a basic method for reducing the risk of potential microbiological hazards and plays an important role in maintaining pool water quality [9,10]. These predictors of risk are being used as the main parameters in water-management programs to prevent and control the growth and spread of *Legionella* [11,12]. Previous studies also suggested that there may be a link between certain water-quality parameters (temperature, free residual chlorine (FRC), pH, etc.) and *L. pneumophila* positivity in water supply systems [13,14,15]. The most probable way that *Legionella* enters public recreational facilities (such as swimming pools, hot tubs, children’s pools, etc.) is through the water-supplying plumbing systems [16]. *L. pneumophila* was considered unable to persist in saline waters, but there are now numerous reports that strongly suggest that *Legionella* spp. could survive in saline environments within certain physicochemical conditions [6,17,18,19]. Therefore, guidelines that focus primarily on building water-supply systems, healthcare facilities, hotels, camps, marinas, cooling towers, cruise ships, and swimming pools must be issued by countries around the world to prevent the occurrence of Legionellosis [20,21]. To improve the quality of water intended for human consumption, a new EU Directive includes this *Legionella* spp. bacterium in routine preventive monitoring programs and must be implemented into the national legislation of the member states by 2023 [22]. Considering all the above-mentioned characteristics, the most direct means of determining the presence or absence of *L. pneumophila* is testing pool water for this microbiological parameter. This study aimed to analyze data on the presence of *L. pneumophila* in recreational swimming pool environments in Primorje-Gorski Kotar County, Croatia. Additionally, the second aim was to find the relationship between *Legionella* positivity with water physicochemical parameters and determine the effectiveness of water-disinfection procedures.

## 2. Materials and Methods

### 2.1. Study Area and Sampling

Swimming pool water samples in Primorje-Gorski Kotar County, Croatia, were analyzed over four years (2018–2022) for *Legionella pneumophila* occurrence and standard physicochemical parameters. Pool waters were sampled once a year, approximately at the same time, and if some of the monitored parameters were not in compliance with the law, sampling was repeated and processed for the incorrect parameter. Water samples were taken in clean containers with a volume of 0.25, 0.5, or 1 L, depending on the analyzed parameter (physicochemical or microbiological). The sampling point was at least 30 cm below the water surface and 30 cm from the pool’s edge using a telescopic rod. Samples were transported to the laboratory in containers with controlled temperature conditions, and if it was impossible to perform a laboratory test immediately, the samples were stored at 4 °C until analysis for a maximum of 24 h.

### 2.2. Measurement of Legionella pneumophila in Pool Water

The presence of *Legionella pneumophila* in pool waters was tested using the membrane filtration technique according to the ISO 11731:2000 standard method [23] as previously described [24]. Filtration of the pool water sample was performed through a 0.2 μm polycarbonate membrane filter 47 mm in diameter (Pall Corporation, Ann Arbor, MI, USA). The membrane was then cut into pieces using sterile scissors to aid elution and transferred to a screw cap sterile container with 10 mL distilled water. To dislodge the microorganisms from the membrane filter, it was vortexed for at least 2 min. A volume of 0.1 mL of the heat-treated sample was inoculated on a selective glycine vancomycin polymyxin B cycloheximide (GVPC) agar plate (OXOID, Basingstoke, UK), incubated for up to 10 days at 36 ± 1 °C in a humid atmosphere with 2.5% CO_2_ and examined every 2–3 days. Each suspicious colony was subcultured on buffered charcoal yeast extract agar (BCYE) and buffered charcoal yeast extract without L-cysteine agar (BCYE-Cys; OXOID, Basingstoke, UK), and incubated at 36 ± 1 °C for >2 days. A commercially available agglutination test (DrySpot *Legionella* Latex Test, Oxoid, UK) was used to differentiate between *L. pneumophila* serogroup 1 and *L. pneumophila* serogroup 2–14. The detection limit of the described procedure was 10 CFU/L.

### 2.3. Physicochemical Parameter Measurements in Pool Water

Selected physicochemical parameters of the pool water, in accordance with the relevant ISO methods, were tested. Water temperature was measured in accordance with Standard Methods 23rd. Ed, 2017; 2550 B [25]. The free residual chlorine (FRC) concentration was measured in accordance with ISO 7393-2:2018 [26]. pH was measured using a multi-channel, modular instrument SevenExcellence (S47, Mettler Toledo, Darmstadt, Germany) according to ISO 10523:2008 [27]. Trihalomethanes (THM) were measured in accordance with ISO 10301:1997 [28].

### 2.4. Statistical Analysis

Descriptive analysis and statistical calculations were performed using Statistica v. 14.0 software (StatSoft Inc., Tulsa, OK, USA) at a significance level of *p* < 0.05. The normality of the experimental data distribution was tested using the Kolmogorov–Smirnov test. Since the obtained distribution was not normal, non-parametric statistical tests were applied to the analysis. All experimental data are expressed as median with the corresponding minimum, maximum, and extreme and outlier values. The effect of the physicochemical parameters of the water in swimming pools (temperature, pH, THM, FRC) on the bacteria *Legionella pneumophila* was studied by the non-parametric Kendall Tau’s correlation test and multiple linear regression test. The distribution of analyzed physicochemical swimming pool parameters and bacteria depending on the type of water in the swimming pools (saltwater or freshwater), pool conditions (outdoor or indoor), and type of swimming pools (children’s, recreational, hot tubs, or sports/Olympic) was determined using the principal component analysis (PCA) test.

## 3. Results and Discussion

Of the total number of samples (N = 4587), 28% were collected in 2018 (N = 1286), 31.7% in 2019 (N = 1454), 16.4% in 2020 (N = 752), and 23.9% in 2021 (N = 1095). Furthermore, most samples contained freshwater from pools (N = 3109; 67.8%), while the rest, 32.2% (N = 1478), contained saltwater from pools. Of the total number of samples, 51.3% were collected from indoor pools (N = 2354), and the rest, 48.7%, were from outdoor pools (N = 2233). Pool water samples were collected from four different types of swimming pools: recreational pools (N = 2833; 61.76%), hot tubs (N = 966; 21.07%), sport/Olympic pools (N = 293; 6.38%), and children’s pools (N = 495; 10.79%).

### 3.1. Microbiological and PhysicoChemical Parameters Measured in Swimming Pool Water Samples

To perform good water management, some public health agencies, as well as guidance documents and new EU directives, recommended monitoring the temperature and water-quality parameters as part of *Legionella* risk assessment management [29]. Following these recommendations, there is a reasonable assumption that there is some relationship between *Legionella* occurrence and certain water-parameter conditions in water environments in general [11]. Monitoring of these parameters in water-supply systems is well described [12,30], but data about Legionella’s presence in recreational swimming pools are comprehensive [31].

**Figure 1:**
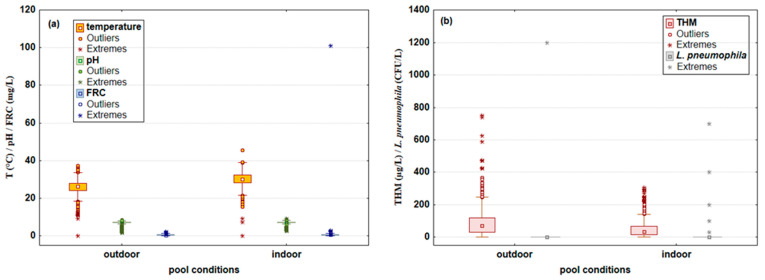
Water’s physicochemical parameters: (**a**) temperature (°C), pH and free residual chlorine (FRC; mg/L), and (**b**) trihalomethanes (THM; μg/L) and amount of *Legionella pneumophila* (CFU/L) in outdoor and indoor swimming pools. Results are shown as median, minimum, and maximum values, with outliers and extreme values (N_pool data_ = 4587; N_outdoor data_ = 2233; N_indoor data_ = 2354).

The distribution of the analyzed water’s physicochemical parameters—the temperature (°C), pH, THM (μg/L), FRC (mg/L), and the number of *L. pneumophila* colonies (CFU/L) in open and indoor swimming pools—is presented in Figure 1a,b.

The median temperature values in outdoor pools were 26.3 °C (max value 37.3 °C) and did not significantly differ from the values obtained for indoor pools of 30.0 °C (max value of 45.5 °C). Very similar behavior was observed for pH, with a median value in outdoor and indoor pools of 7.3 and 7.2, respectively. It should be noted that the pH value oscillations were significant and ranged from 1.6 to 8.7 in outdoor pools and from 2.5 to 9.2 in indoor pools. Furthermore, the FRC median values in both types of pools did not differ significantly (0.56 mg/L in outdoor and 0.60 mg/L in indoor pools), but some more extreme values were found in indoor pools, amounting to 2.80 mg/L compared to indoor pools, where a maximum value of 2.20 mg/L was reached. A somewhat different behavior was observed for the THM amount, where significantly higher values were found in outdoor pools (median 70 μg/L) compared to indoor pools, with median values of 33 μg/L. Furthermore, more extreme THM values were obtained in outdoor pools, ranging from 422 to 751 μg/L, while the highest THM value in indoor pools was 308 μg/L. The bacterium *L. pneumophila*’s SG 1 presence was proven in a total of six pool water samples. Only one was an outdoor pool sample (1200 CFU/L), while five positive samples were found in indoor pools, with values ranging from 30 to 700 CFU/L.

**Figure 2:**
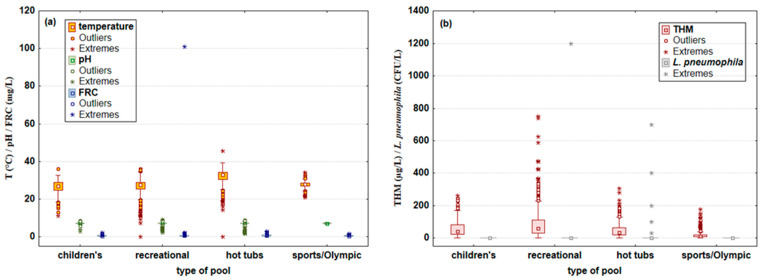
Water physicochemical parameters: (**a**) temperature (°C), pH, and free residual chlorine (FRC; mg/L), and (**b**) trihalomethanes (THM; μg/L) and amount of *Legionella pneumophila* (CFU/L) in children’s, recreational, hot tubs, and sports/Olympic swimming pools. Results are shown as median, minimum, and maximum values with outliers and extreme values (N_pool data_ = 4587; N_children’s data_ = 495; N_recreational data_ = 2833; N_hot tubs data_ = 966; N_sport/Olympic data_ = 293).

The distribution of the same parameters, depending on the type of pool (children’s, recreational, hot tubs, or sports/Olympic pools), is presented in Figure 2a,b.

As expected, the highest values of pool water temperature were detected in hot tubs (33.0 °C), while the average temperature values in other pool types were 27.4 °C. Median pH values in all pool types were 7.3, with a note that oscillations were evident in all pool types from 1.6 to 9.2, except for sports/Olympic pools, where the variation was less pronounced (6.6 to 7.7). The median FRC values were approximately similar and amounted to ≈0.58 mg/L in all pool types, with the lowest median FRC value detected in sport/Olympic pools (0.44 mg/L), where a lower maximum value was found (1.58 mg/L) compared to other pool types (2.8 mg/L). THM median values ranged from 12.5 μg/L (sport/Olympic) to 62 μg/L (recreational). Extreme THM values (above 400 μg/L) were found in six samples of recreational pools. The presence of the bacterium *L. pneumophila* was proven in five samples of hot tub pool water (30 to 700 CFU/L) and in one sample from a recreational pool (1200 CFU/L).

**Figure 3:**
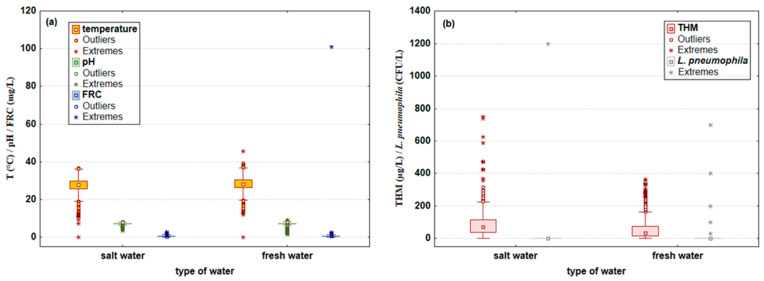
Water physicochemical parameters: (**a**) temperature (°C), pH, and free residual chlorine (FRC; mg/L), and (**b**) trihalomethanes (THM; μg/L) and amount of *Legionella pneumophila* (CFU/L) in swimming pools with fresh and salt water. Results are shown as median, minimum, and maximum values with outliers and extreme values (N_pool data_ = 4587; N_saltwater data_ = 1478; N_freshwater data_ = 3109).

Finally, the physicochemical parameters and the presence of *L. pneumophila* in pools filled with freshwater and saltwater were also studied and are presented in Figure 3a,b.

Median temperature values were approximately the same in both pool types (≈28 °C), with a higher maximum temperature found in pools filled with fresh water (45.5 °C) compared to pools with salt water (36.7 °C). Very similar behavior was also achieved with pH values and FRC concentration, with median pH and FRC values of 7.3 and 0.6 mg/L, respectively, with slightly higher maximum pH values determined in pools with fresh water (9.2), while the maximum FRC values for both pool types were identical and amounted to 2.8 mg/L. An obvious difference was observed in the presence of THMs, where much higher concentrations were found in pools with salt water (median 71 μg/L), while values of 34 μg/L were detected in pools with fresh water. Furthermore, it should be noted that extreme THM values >400 μg/L (five samples) were also found in pools with salt water. The presence of *L. pneumophila* SG 1 in five fresh pool water samples and only one with salt water was determined. Based on the facts presented, it can be concluded that of the total pool water samples analyzed (N = 4587), *L. pneumophila* was detected in six of them, representing 0.1% positive samples. Five positive samples were found in the water of indoor hot tubs filled with freshwater (0.5% positive samples), while only one positive sample was found in an outdoor recreational saltwater pool (less than 0.1% positive samples).

Our results are similar to those obtained in the assessment of the water quality of some swimming pools in Alexandria, Egypt, where temperature data ranged between 25.2 °C and 33.1 °C [32]. These findings are also consistent with another study in Bologna, Italy, where the temperature range was between 25.2 °C and 33.1 °C [33]. Some guidelines provide specific sites that are potential sources of *Legionella*, but in the case of swimming pool facilities, many should be further investigated in separate assessments. It is expected that swimming pools with a water temperature higher than 27 °C are more likely to be contaminated with pathogens than those below 27 °C because a higher water temperature can promote the faster growth of some microorganisms [34]. The occurrence of *L. pneumophila* at a higher rate in indoor pools than in outdoor is expected since there are fewer temperature fluctuations due to less air flow in indoor interiors. Our results on the presence of *Legionella* spp. in hot tubs and recreational pool water comply with other previously reported investigations where these pools were the major sources of both Pontiac fever and Legionnaire’s disease [35,36]. We also confirmed in our previous field investigation that hot tub water was also associated with a single case of Legionnaire’s disease. In that case, it was proven that the source of *Legionella* contamination was tap water used to fill up the hot tub [6]. There were no reported LD cases during the survey. Higher values of FRC in combination with organic matter almost always bring higher values of THM. Regarding the physicochemical parameters in water, more severe consequences are related to low values of FRC compared to high values in some pool water samples [37]. A somewhat lower range of THM results was presented by Silva et al. in a Portuguese study, where THM ranged from 10 to 155 μg/L and, as reported by Canadian researchers, 17.5–113.5 μg/L [37,38]. Depending on the pool water disinfection method used, the production of by-products such as THMs can be significantly reduced [39]. The pH is a very important parameter since maintaining it in the correct range ensures proper disinfection and a microbiologically and chemically safe water environment [40]. It is of great interest to develop a risk-management procedure where possible to predict the risk of infection when exposed to certain *L. pneumophila* bacteria counts. Bouwknegt et al. estimated that the risk of *L. pneumophila* infection ranged from 3% to >95% (bacterial count of 10 and ≥1000 CFU/L, respectively) after a 15 min stay in an active whirlpool [41]. The Croatian Regulation on Health Safety of Swimming Pool Water (OG 59/2020), among other parameters, prescribes that the pH levels in pools should be maintained between 6.5 and 7.3 for freshwater and seawater, FRC should be no more than 1.2 mg/L Cl_2_, and the highest acceptable level of THMs is 100 µg/L [9,42]. The obtained results regarding FRC, pH and THMs were somewhat higher or lower than the Regulation suggests which affected the overall opinion of the analytical report created for the pool owners.

### 3.2. Predictive Models for the Incidence of Legionella pneumophila

Furthermore, to assess the influence of the physicochemical parameters of the analyzed pool waters on the presence of *L. pneumophila* in more detail, Kendall’s Tau correlation analysis was used, the results of which are shown in Table 1.

The results are presented only for conditions and pool types where the specified bacteria was detected (outdoor and indoor pools, recreational pools and hot tubs, and pools filled with saltwater and freshwater). The correlation analysis of the pooled data of outdoor swimming pools (N = 2233) indicated a positive and statistically significant correlation between the presence of *L. pneumophila* and temperature (τ = 0.03), while the correlations with the remaining parameters were negative and not statistically significant, which proves the dominant role of temperature on the presence of bacteria in outdoor pools. The temperature, pH, and concentration of FRC had a positive effect on the presence of *L. pneumophila* in indoor pools; however, this influence was not statistically significant (N = 2354). Almost identical behavior, i.e., the dominance of the temperature influence (τ = 0.03) on the presence of *L. pneumophila* bacteria, was observed in recreational pools (N = 2833), while in hot tubs, the strongest influence on the presence of bacteria was due to FRC and pH, but these effects were not statistically significant (N = 966). The correlational analysis of the saltwater pool’s physicochemical parameters and the presence of *L. pneumophila* indicated the positive and statistically significant influence of temperature (τ = 0.03; N = 1478). In the freshwater pool, the strongest positive and statistically significant effect was due to temperature (τ = 0.04; N = 3109), although pH and FRC showed positive, somewhat weaker effects on the presence of bacteria. The correlations of the physicochemical parameters of the pool water and the presence of *L. pneumophila* are also shown graphically, the results of which are presented in Figure S1a–f. It is noticeable that the only statistically significant correlation (*p* = 0.037) was achieved between temperature and *L. pneumophila* in pools with fresh water. Furthermore, the dependence of temperature on other physicochemical parameters (pH, THM and FRC) was analyzed and the results are presented graphically in Figure S2a–f. The statistically significant dependence of temperature on all the pool water physicochemical parameters was achieved in outdoor pools (with pH, *p* < 0.001; with THM, *p* = 0.016; with FRC, *p* < 0.001) and hot tubs (with pH, *p* = 0.013; with THM, *p* = 0.004; with FRC, *p* < 0.001). In swimming pools with freshwater, a statistically significant effect was achieved between temperature and parameters: pH (*p* < 0.001), and THM (*p* < 0.001), in pools with saltwater between temperature and FRC (*p* < 0.001), and in recreational swimming pools between temperature and THM (*p* = 0.032).

Since the pool water parameters can significantly influence the growth and presence of bacteria, especially the analyzed *L. pneumophila*, there is a serious need to determine the simultaneous effects of all analyzed physicochemical parameters on the presence of *L. pneumophila* in pool water. For this reason, a predictive model that shows the simultaneous influence of chemical parameters on the incidence of *L. pneumophila* can have a significant impact. Therefore, in addition to the non-parametric Kendall’s Tau test, a multiple linear regression test was also used. This test simultaneously compares changes in different variables—in this case, the physicochemical parameters of the analyzed pool waters—and their simultaneous influence on the presence of *L. pneumophila*, thus helping to form a linear predictive model of water contamination with bacteria. Multiple linear regression resulted in the correlations listed below, where bold letters and numbers indicate statistically significant correlations with *p* < 0.05.

$$L.pneumophila_{outdoor}=0.31\times T-0.16\times pH-0.01\times THH-0.84\times FRC-4.89;\;R^{2}=0.65$$

$$L.pneumophila_{indoor}=0.17\times T+0.38\times pH-0.01\times THH+0.01\times FRC-6.90;\;R^{2}=0.36$$

$$L.pneumophila_{recreational}=0.19\times T-0.29\times pH-0.01\times THH-0.03\times FRC-2.17;\;R^{2}=0.45$$

$$L.pneumophila_{hot\;tubs}=0.09\times T+0.63\times pH-0.02\times THH+1.19\times FRC-5.67;\;R^{2}=0.39$$

$$L.pneumophila_{salt\;water}=0.27\times T-1.41\times pH-0.01\times THH-1.78\times FRC+5.78;\;R^{2}=0.40$$

$$L.pneumophila_{fresh\;water}=0.14\times T+0.29\times pH-0.01\times THH+0.01\times FRC-5.57;\;R^{2}=0.56$$

In two out of three cases, the results of multiple regression confirmed the conclusions obtained by Kendall’s Tau correlation about the importance of temperature on the occurrence of *L. pneumophila*. However, although Kendall’s Tau correlation of pooled data for hot tubs indicated that temperature has no significant effect on the presence of *L. pneumophila*, multiple regression analysis showed that a positive temperature contribution could be expected in all analyzed samples. Furthermore, it is also noticeable that both pH and FRC played an important role as growth parameters of *L. pneumophila*.

**Figure 4:**
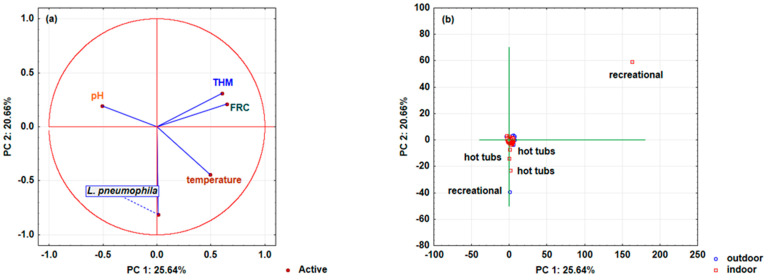
Distribution of analyzed swimming pool water’s physicochemical parameters (temperature (°C), pH, trihalomethanes (THM; μg/L), free residual chlorine (FRC; mg/L)), and bacteria: *Legionella pneumophila* (CFU/L), depending on whether it was an indoor or outdoor swimming pool and the type of swimming pool (children’s, recreational, hot tub, or sports/Olympic), as determined by principal component analysis (PCA) with main components PC 1 and PC 2. Projections of (**a**) the variables (swimming water pools’ physicochemical parameters and bacteria) and (**b**) cases (type of swimming pools and outdoor or indoor conditions) on the factor plane. (N_pool data_ = 4587; N_outdoor data_ = 2233; N_indoor data_ = 2354).

**Figure 5:**
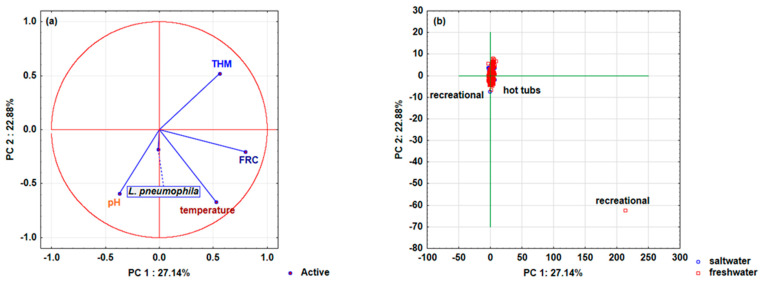
Distribution of analyzed swimming pool water’s chemical parameters (temperature (°C), pH, trihalomethanes (THM; μg/L), free residual chlorine (FRC; mg/L)), and bacteria: *Legionella pneumophila* (CFU/L) depending on water in the swimming pool (saltwater or freshwater) and type of swimming pool (children’s, recreational, hot tub, or sports/Olympic), represented by principal component analysis (PCA) with main components PC 1 and PC 2. Projections of (**a**) the variables (swimming pool water’s chemical parameters and bacteria) and (**b**) cases (type of swimming pools and water in the swimming pools) on the factor plane. (N_pool data_ = 4587; N_saltwater data_ = 1478; N_freshwater data_ = 3109).

Finally, the analyzed swimming pools’ chemical parameter variable distribution (temperature, pH, THM, FRC, and *L. pneumophila*) depending on pool conditions (indoor or outdoor), the type of pool (children’s, recreational, hot tubs, or sports/Olympic) (Figure 4a,b), and the dependence on freshwater and saltwater (Figure 5a,b) is presented by principal component analysis (PCA).

Three main components (PC 1, PC 2, and PC 3 according to Cattell’s scree test) were retained in the analysis, explaining 65.31% (for indoor or outdoor swimming pools) and 70.09% (for swimming pools with freshwater and saltwater), it was found that PC1 explains 25.64% of the total variance, PC 2 explains 20.66%, and PC 3 explains 19.01%, while the analysis of freshwater and saltwater pools with PC1, PC2, and PC3 explained 27.14, 22.88, and 20.07% of the total variance, respectively. Figure 4a shows the distribution of the analyzed pool water’s chemical parameters and the presence of *L. pneumophila* depending on the conditions (outdoor or indoor pool) and pool types. Most of the analyzed variables (*L. pneumophila*, temperature, FRC, and THM) are located in the first and second quadrants and define the positive side of the main component PC 1 (right side of PC 1), while the variable pH is in the third quadrant and defines the negative side of the main component PC 1 (left page of PC 1). None of the analyzed variables were found in the remaining fourth quadrant. Furthermore, the importance of the analyzed variables within the main components in the model definition was determined (Table S1). The strongest influence on the PC 1 component definition is shown by FRC (0.33), while the influence of THM (0.29) and temperature (0.19) is slightly less pronounced. The variable *L. pneumophila* defines the main component PC 2 (0.64), while pH (0.37) only defines the main component PC 3. Analyzing the pool types’ distribution depending on the conditions (outdoor or indoor pool) represented by the main components PC 1 and PC 2 (Figure 4b), it is evident that the variables of the indoor hot tubs and one variable of the recreational outdoor pool are distributed on the axis of the main component PC 1, leading to assumptions that *L. pneumophila* was detected in these pools. The most important parameter for the presence of *L. pneumophila* in these pools is temperature, but the presence of FRC and THM is also evident, while the influence of the water’s pH is less pronounced.

Somewhat different behavior was observed where the effect of fresh and salt pool water was analyzed. In this case, the distribution of variables on the dependence of the main components PC 1 and PC 2 is shown since most of the variables significantly contribute to the model’s definition using these two main components (Figure 5a). FRC and temperature are located in the first quadrant and only the variable THM is located in the second quadrant and all the mentioned variables define the positive side of the main component PC 1. Variables *L. pneumophila*, and pH are located in the fourth quadrant and define the negative side of the main component PC 1. Analyzing the variables’ importance in the model definition (Table S2), the strongest influence of temperature (0.39) on the definition of the PC 2 component was found, along with a somewhat weaker effect of pH (0.31) and THM (0.24), while the influence of FRC was almost negligible (0.04). The contribution of the *L. pneumophila* variable in defining the main component PC 3 is significant and amounts to 0.93, while the FRC contributes to PC1 with 0.47. Figure 5b shows the distribution of pool types depending on the type of pool water (freshwater, saltwater) and the main components PC 1 and PC 2, where the presence of *L. pneumophila* can be observed in hot tubs with freshwater and in one recreational pool with saltwater. In this case, the presence of *L. pneumophila* in these pools is dominated by temperature and pH. The results obtained in this study are opposite to those reported in an American study where there was no statistically significant correlation between distal site *Legionella* positivity and the temperature, pH, and FRC of water samples [29]. It was challenging to design the presented survey with a hypothesis that the certain connection could be established between the presence of *L. pneumophila* and the physicochemical parameters in a representative number of pool water samples, by statistical methods. The obtained results present temperature as a significant parameter from a statistical point of view, but PCA analysis demonstrated that all parameters had an influence.

Even though we found some positive correlation between the physicochemical parameters of water and the presence of *L. pneumophila*, we can conclude that regular measurements cannot predict the presence or absence of *Legionella* species in water risk management. We have to be aware that many Legionnaire’s disease cases remain unknown and are not linked to the place of infection. Therefore, to develop better preventive measures, we need stricter requirements for a legally allowed minimum *Legionella* concentration in bathing water [43].

## 4. Conclusions

After confirmed laboratory research, the correction measures regarding poor water disinfection were implemented by the technical experts and supervised by environmental health professionals.

It is necessary to keep all pool water physicochemical parameters in perfect harmony to reach the best disinfection procedure and to avoid conditions for *Legionella pneumophila* occurrence.

Predictive models from the statistical point of view showed that temperature is the most important parameter determining the presence of *L. pneumophila* in pool water.

Temperatures between 20 and 45 °C can induce *L. pneumophila* to multiply; it is usually dormant below 20 °C, and does not survive above 60 °C.

## Figures and Tables

**Table 1:** Correlation coefficients between physicochemical parameters of analyzed swimming water pools (temperature (°C), pH, trihalomethanes (THM; μg/L), free residual chlorine (FRC; mg/L), and *Legionella pneumophila* (CFU/L) and different pool characteristics (outdoor, indoor, recreational, hot tubs, saltwater, and freshwater), represented by non-parametric Kendall’s Tau correlation test. Statistically significant correlations (*p* < 0.05) are presented with bold type numbers.

Pool Characteristics	Variable	Temperature (°C)	pH	THM (μg/L)	FRC (mg/L)
Outdoor swimming pools (N = 2233)	*Legionella pneumophila* (CFU/L)	**0.03**	−0.01	−0.03	−0.01
Indoor swimming pools (N = 2354)		0.02	0.01	−0.01	0.01
Recreational (N = 2833)		**0.03**	−0.01	−0.03	−0.01
Hot tubs (N = 966)		−0.02	0.01	−0.02	0.02
Saltwater (N = 1478)		0.03	−0.02	−0.03	−0.01
Freshwater (N = 3109)		**0.04**	0.01	−0.01	0.01

## Data Availability

The data supporting the findings of this study are available within the article or the Supplementary Material.

## Supplementary Materials

The following supporting information can be downloaded at: https://www.mdpi.com/article/10.3390/microorganisms10122429/s1, Figure S1: Correlations between *L. pneumophila* and physico-chemical characteristics (temperature, pH, THM, FRC) of waters in: (a) outdoor, (b) indoor, (c) recreational swimming pools, (d) hot tubs, (e) swimming pools with salt water, and (f) swimming pools with fresh water; Figure S2: Correlations between temperature and physico-chemical characteristics (pH, THM, FRC) of waters in: (a) outdoor, (b) indoor, (c) recreational swimming pools, (d) hot tubs, (e) swimming pools with salt water, and (f) swimming pools with fresh water; Table S1: Eigenvector values and variable contributions/indoor or outdoor swimming pool and the type of swimming pool; Table S2: Eigenvector values and variable contributions/saltwater or freshwater and type of swimming pool.

## References

[1] Leoni E., Catalani F., Marini S., Dallolio L. (2018). Legionellosis Associated with Recreational Waters: A Systematic Review of Cases and Outbreaks in Swimming Pools, Spa Pools, and Similar Environments. Int. J. Environ. Res. Public Health.

[2] Chambers S.T., Slow S., Scott-Thomas A., Murdoch D.R. (2021). Legionellosis Caused by Non-*Legionella Pneumophila* Species, with a Focus on *Legionella Longbeachae*. Microorganisms.

[3] European Centre for Disease Prevention and Control (2020). Legionnaires’ disease. Annual Epidemiological Report for 2018.

[4] Del Piano M., La Palombara P., Nicosia R., Picchiotti R. (1984). The legionellosis. Boll Ist Sieroter Milan..

[5] Ryu S., Yang K., Chun B.C. (2017). Community-acquired Legionnaires’ Disease in a Newly Constructed Apartment Building. Prev. Med. Public Health.

[6] Tomić Linšak D., Keše D., Broznić D., Vukić L.D., Cenov A., Morić M., Gobin I. (2021). Sea water whirlpool spa as a source of *Legionella* infection. J. Water Health.

[7] Assaidi A., Ellouali M., Latrache H., Zahir H., Mliji E.M. (2021). Role of Biofilms in the Survival of *Legionella Pneumophila* to Sodium Chloride Treatment. Iran J. Microbiol..

[8] Iliadi V., Staykova J., Iliadis S., Konstantinidou I., Sivykh P., Romanidou G., Vardikov D.F., Cassimos D., Konstantinidis T.G. (2022). *Legionella pneumophila*: The Journey from the Environment to the Blood. J. Clin. Med..

[9] Sigler Zekanović M., Begić G., Medić A., Gobin I., Tomić Linšak D. (2022). Effects of a Combined Disinfection Method on *Pseudomonas aeruginosa* Biofilm in Freshwater Swimming Pool. Environments.

[10] Giampaoli S., Spica V.R. (2014). Health and safety in recreational waters. Bull. World Health Organ..

[11] Ditommaso S., Giacomuzzi M., Gentile M., Moiraghi A.R., Zotti C.M. (2010). Effective environmental sampling strategies for monitoring *Legionella* spp contamination in hot water systems. Am. J. Infect. Control.

[12] Bédard E., Fey S., Charron D., Lalancette C., Cantin P., Dolcé P., Laferrière C., Déziel E., Prévost M. (2015). Temperature diagnostic to identify high risk areas and optimize *Legionella pneumophila* surveillance in hot water distribution systems. Water Res..

[13] Wright D.R., Centers for Medicare & Medicaid Services (2017). Requirement to Reduce Legionella Risk in Healthcare Facility Water Systems to Prevent Cases and Outbreaks of Legionnaires’ Disease (LD). S&C 17-30-Hospitals/CAHs/NHs.

[14] Centers for Disease Control and Prevention (CDC) (2017). Developing a Water Management Program to Reduce Legionella Growth & Spread in Buildings—A Practical Guide to Implementing Industry Standards.

[15] Borella P., Montagna M.T., Romano-Spica V., Stampi S., Stancanelli G., Triassi M., Neglia R., Marchesi I., Fantuzzi G., Tato D. (2004). *Legionella* infection risk from domestic hot water. Emerg. Infect. Dis..

[16] Rakic A., Vukic Lušic D., Jurčev Savicević A. (2022). Influence of Metal Concentration and Plumbing Materials on Legionella Contamination. Microorganisms.

[17] Ortiz-Roque C.M., Hazen T.C. (1987). Abundance and distribution of Legionellaceae in Puerto Rican waters. Appl. Environ. Microbiol..

[18] Palmer C.J., Tsai Y.L., Paszko-Kolva C., Mayer C., Sangermano L.G. (1993). Detection of Legionella species in sewage and ocean water by polymerase chain reaction direct fluorescent antibody and plate culture methods. Appl. Environ. Microbiol..

[19] Heller R., Höller C., Süssmuth R., Gundermann K.O. (1998). Effect of salt concentration and temperature on survival of *Legionella pneumophila*. Lett. Appl. Microbiol..

[20] Parr A., Whitney E.A., Berkelman R.L. (2015). Legionellosis on the Rise: A Review of Guidelines for Prevention in the United States. J. Public Health Manag. Pract..

[21] Van Kenhove E., Dinne K., Janssens A., Laverge J. (2019). Overview and comparison of *Legionella* regulations worldwide. Am. J. Infect. Control.

[22] (2020). Directive (EU) 2020/2184 of 16 December 2020 on the Quality of Water Intended for Human Consumption (Recast).

[23] (2017). Water Quality—Enumeration of Legionella.

[24] Glažar Ivče D., Rončević D., Šantić M., Cenov A., Tomić Linšak D., Mićović V., Lušić D., Glad M., Ljubas D., Vukić Lušić D. (2021). Is a Proactive Approach to Controlling *Legionella* in the Environment Justified?. Food Technol. Biotechnol..

[25] (2017). Standard Method 2550 B. Temperature of Water. Standard Methods for the Examination of Water and Waste Water.

[26] (2018). Water Quality—Determination of Free Chlorine and Total Chlorine—Part 2: Colorimetric Method Using N,N-Diethyl-1,4-Phenylenediamine, for Routine Control Purposes.

[27] (2008). Water Quality—Determination of pH.

[28] (1997). Water Quality—Determination of Highly Volatile Halogenated Hydrocarbons—Gas-Chromatographic Methods.

[29] Pierre D., Baron J.L., Ma X., Sidari III F.P., Wagener M.M., Stout J.E. (2018). Water quality as a predictor of *Legionella* positivity of building water systems. Pathogens.

[30] Bargellini A., Marchesi I., Righi E., Ferrari A., Cencetti S., Borella P., Rovesti S. (2011). Parameters predictive of *Legionella* contamination in hot water systems: Association with trace elements and heterotrophic plate counts. Water Res..

[31] Zwiener C., Richardson S.D., DeMarini D.M., Grummt T., Glauner T., Frimmel F. (2007). Drowning in disinfection by-products? Assessing swimming pool water. Environ. Sci. Technol..

[32] Abd El-Salam M.M. (2012). Assessment of water quality of some swimming pools: A case study in Alexandria, Egypt. Environ. Monit. Assess..

[33] Leoni E., Legnani P., Guberti E., Masotti A. (1999). Risk of infection associated with microbiological quality of public swimming pools in Bologna, Italy. Public Health.

[34] World Health Organization (WHO) (2006). Swimming pools and similar environments. Guidelines for Safe Recreational Water Environments.

[35] Kuroki T., Amemura-Maekawa J., Ohya H., Furukawa I., Suzuki M., Masaoka T., Kura F. (2017). Outbreak of Legionnaire’s Disease Caused by *Legionella pneumophila* Serogroups 1 and 13. Emerg. Infect. Dis..

[36] Euser S.M., Pelgrim M., den Boer J.W. (2010). Legionnaires’ disease and Pontiac fever after using a private outdoor whirlpool spa. Scand. J. Infect. Dis..

[37] Silva Z.I., Rebelo M.H., Silva M.M., Alves A.M., Cabral Mda C., Almeida A.C., Aguiar F.R., de Oliveira A.L., Nogueira A.C., Pinhal H.R. (2012). Trihalomethanes in Lisbon indoor swimming pools: Occurrence, determining factors, and health risk classification. J. Toxicol. Environ. Health Part A.

[38] Simard S., Tardif R., Rodriguez M.J. (2013). Variability of chlorination by-product occurrence in water of indoor and outdoor swimming pools. Water Res..

[39] Kudlek E., Lempart A., Dudziak M., Bujak M. (2018). Impact of the UV lamp power on the formation of swimming pool water treatment by-products. Archit. Civ. Eng. Environ..

[40] Marco E., Lourencetti C., Grimalt J.O., Gari M., Fernández P., Font-Ribera L., Villanueva C.M., Kogevinas M. (2015). Influence of physical activity in the intake of trihalomethanes in indoor swimming pools. Environ. Res..

[41] Bouwknegt M., Schijven J.F., Schalk J.A., de Roda Husman A.M. (2013). Quantitative risk estimation for a *Legionella pneumophila* infection due to whirlpool use. Risk Anal..

[42] Official Gazette of the Republic of Croatia (2020). Regulation on Sanitary-Technical and Hygienic Conditions of Swimming Pools and on the Health Safety of Pool Waters.

[43] Armstrong T.W., Haas C.N. (2007). Quantitative microbial risk assessment model for Legionnaires’ disease: Assessment of human exposures for selected spa outbreaks. J. Occup. Environ. Hyg..

