# Supplementary material for: Surveillance of Legionella pneumophila: Detection in Public Swimming Pool Environment
Source: Microorganisms. 2022 Dec 8;10(12):2429. doi: 10.3390/microorganisms10122429 (PMC9784426; doi:10.3390/microorganisms10122429)
Supplement: Supplementary file 1 [file microorganisms-10-02429-s001.zip › microorganisms-2057063-supplementary.pdf]

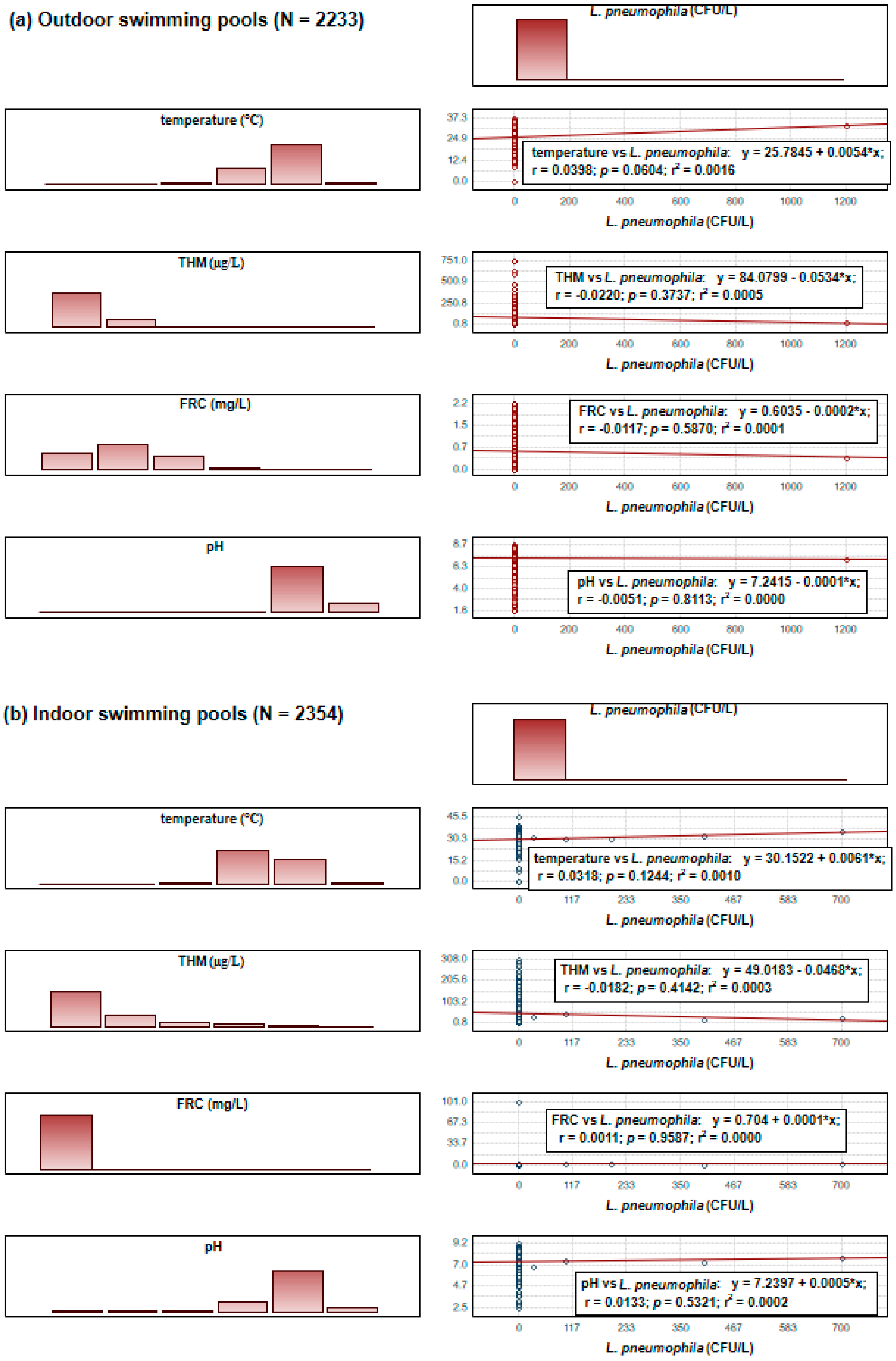
(a) Outdoor swimming pools (N = 2233)
L. pneumophila (CFU/L)
temperature (°C)
temperature vs L. pneumophila: y = 25.7845 + 0.0054*x; r = 0.0398; p = 0.0604; r² = 0.0016
THM (µg/L)
THM vs L. pneumophila: y = 84.0799 - 0.0534*x; r = -0.0220; p = 0.3737; r² = 0.0005
FRC (mg/L)
FRC vs L. pneumophila: y = 0.6035 - 0.0002*x; r = -0.0117; p = 0.5870; r² = 0.0001
pH
pH vs L. pneumophila: y = 7.2415 - 0.0001*x; r = -0.0051; p = 0.8113; r² = 0.0000
(b) Indoor swimming pools (N = 2354)
L. pneumophila (CFU/L)
temperature (°C)
temperature vs L. pneumophila: y = 30.1522 + 0.0061*x; r = 0.0318; p = 0.1244; r² = 0.0010
THM (µg/L)
THM vs L. pneumophila: y = 49.0183 - 0.0468*x; r = -0.0182; p = 0.4142; r² = 0.0003
FRC (mg/L)
FRC vs L. pneumophila: y = 0.704 + 0.0001*x; r = 0.0011; p = 0.9587; r² = 0.0000
pH
pH vs L. pneumophila: y = 7.2397 + 0.0005*x; r = 0.0133; p = 0.5321; r² = 0.0002

(c) Recreational swimming pools (N = 2833)

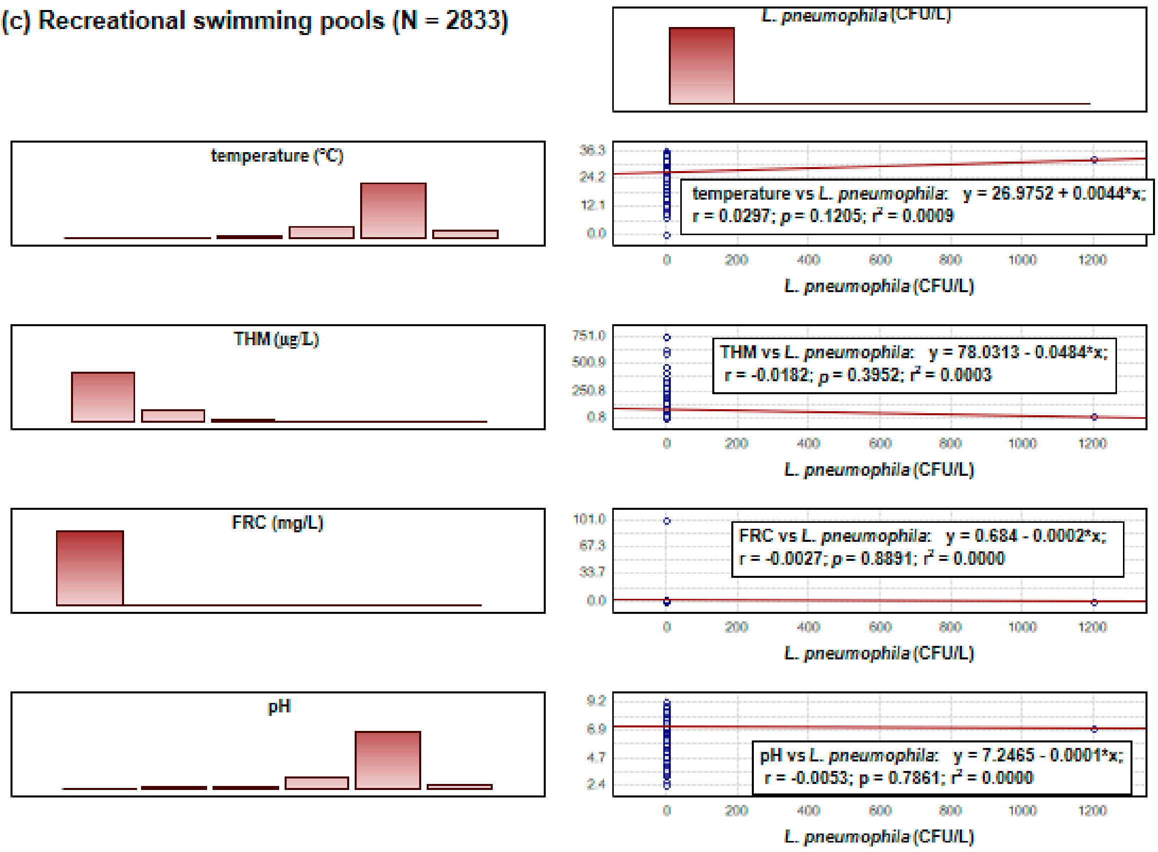

(d) Hot tubs (N = 966)

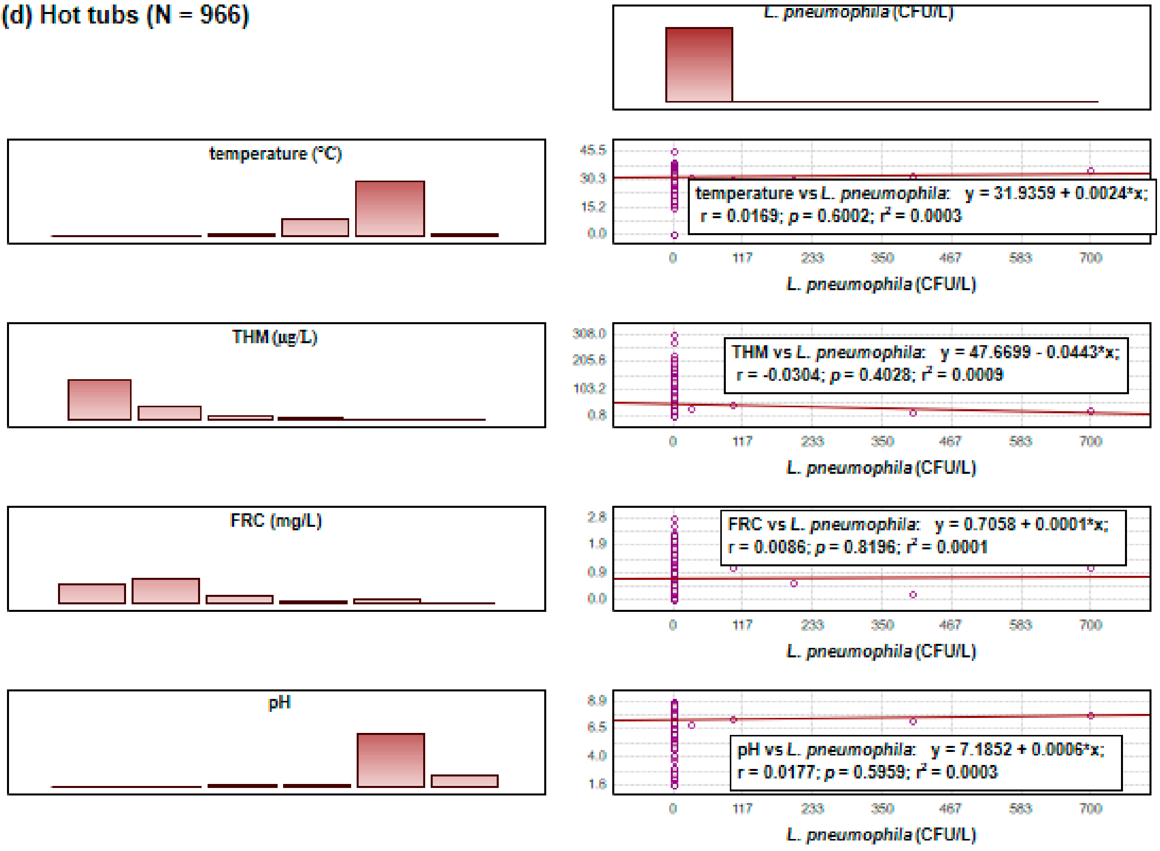

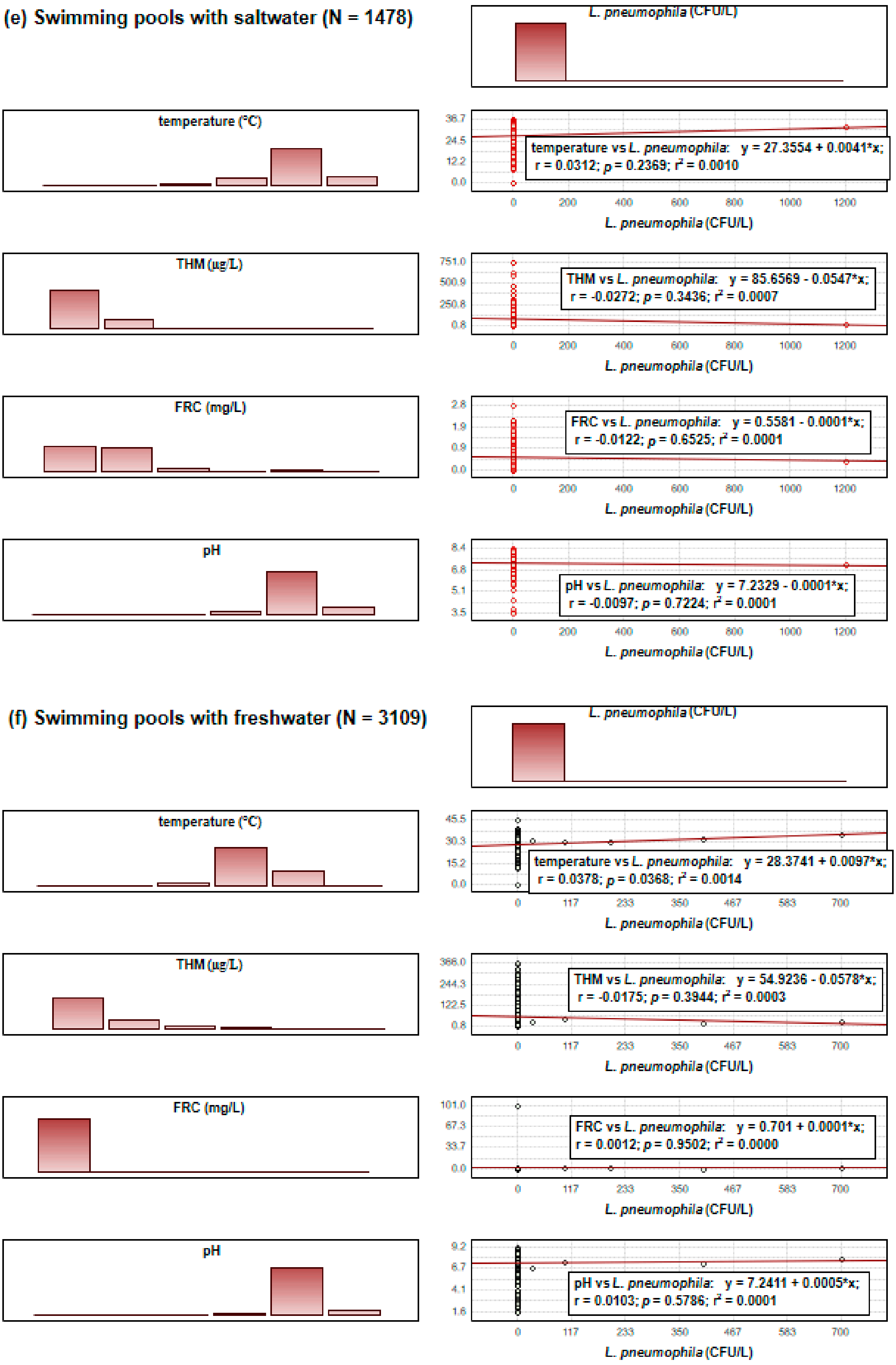

Figure S1. Correlations between *L. pneumophila*and physico-chemical characteristics (temperature, pH, THM, FRC) of waters in: a) outdoor , b) indoor, c) recreational swimming pools, d) hot tubs, e) swimming pools with salt water, and f) swimming pools with fresh water.

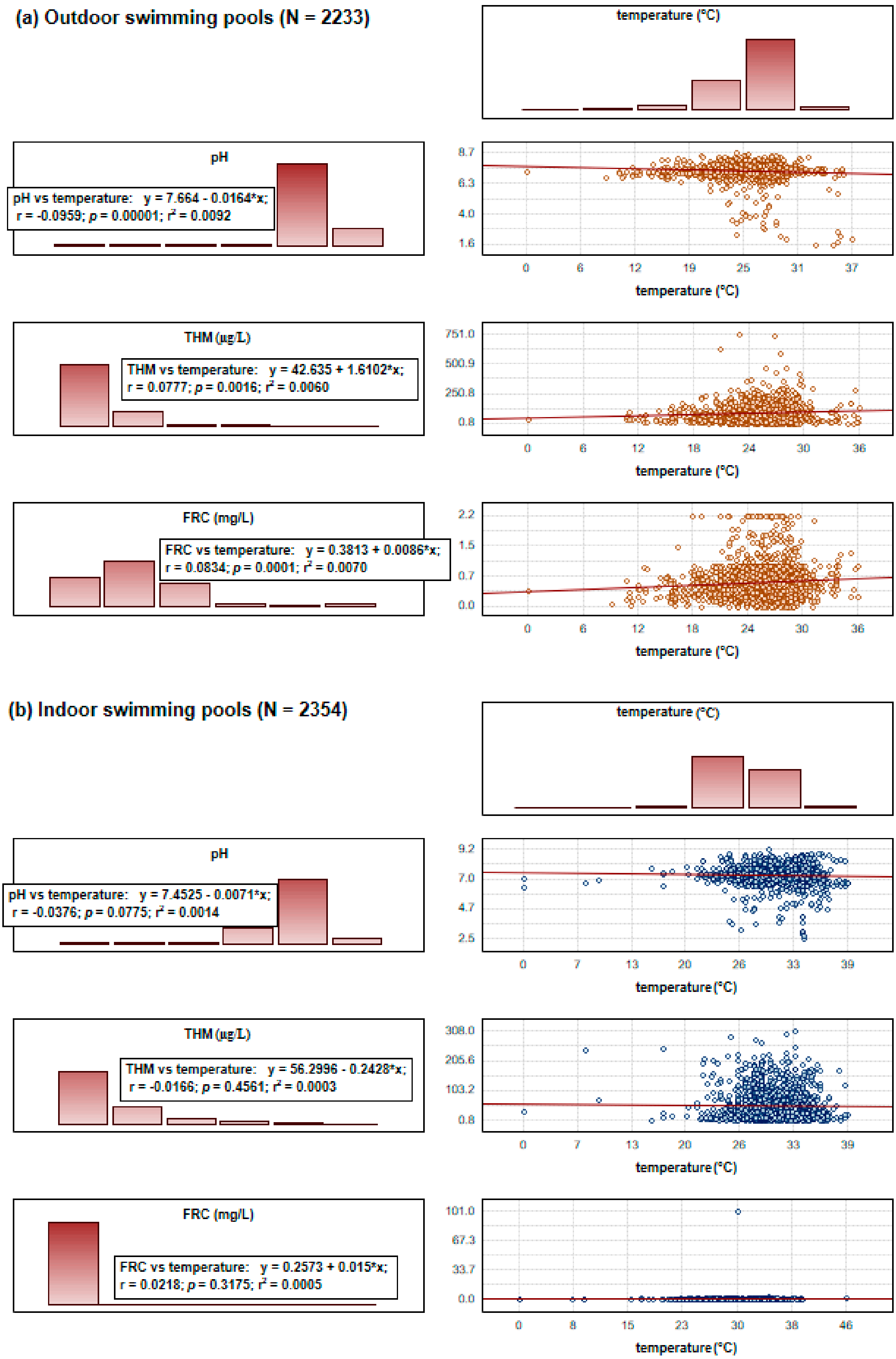
(a) Outdoor swimming pools (N = 2233)
temperature (°C)
pH
pH vs temperature: y = 7.664 - 0.0164*x;
r = -0.0959; p = 0.00001; r² = 0.0092
THM (µg/L)
THM vs temperature: y = 42.635 + 1.6102*x;
r = 0.0777; p = 0.0016; r² = 0.0060
FRC (mg/L)
FRC vs temperature: y = 0.3813 + 0.0086*x;
r = 0.0834; p = 0.0001; r² = 0.0070
temperature (°C)
(b) Indoor swimming pools (N = 2354)
temperature (°C)
pH
pH vs temperature: y = 7.4525 - 0.0071*x;
r = -0.0376; p = 0.0775; r² = 0.0014
THM (µg/L)
THM vs temperature: y = 56.2996 - 0.2428*x;
r = -0.0166; p = 0.4561; r² = 0.0003
FRC (mg/L)
FRC vs temperature: y = 0.2573 + 0.015*x;
r = 0.0218; p = 0.3175; r² = 0.0005
temperature (°C)

(c) Recreational swimming pools (N = 2833)

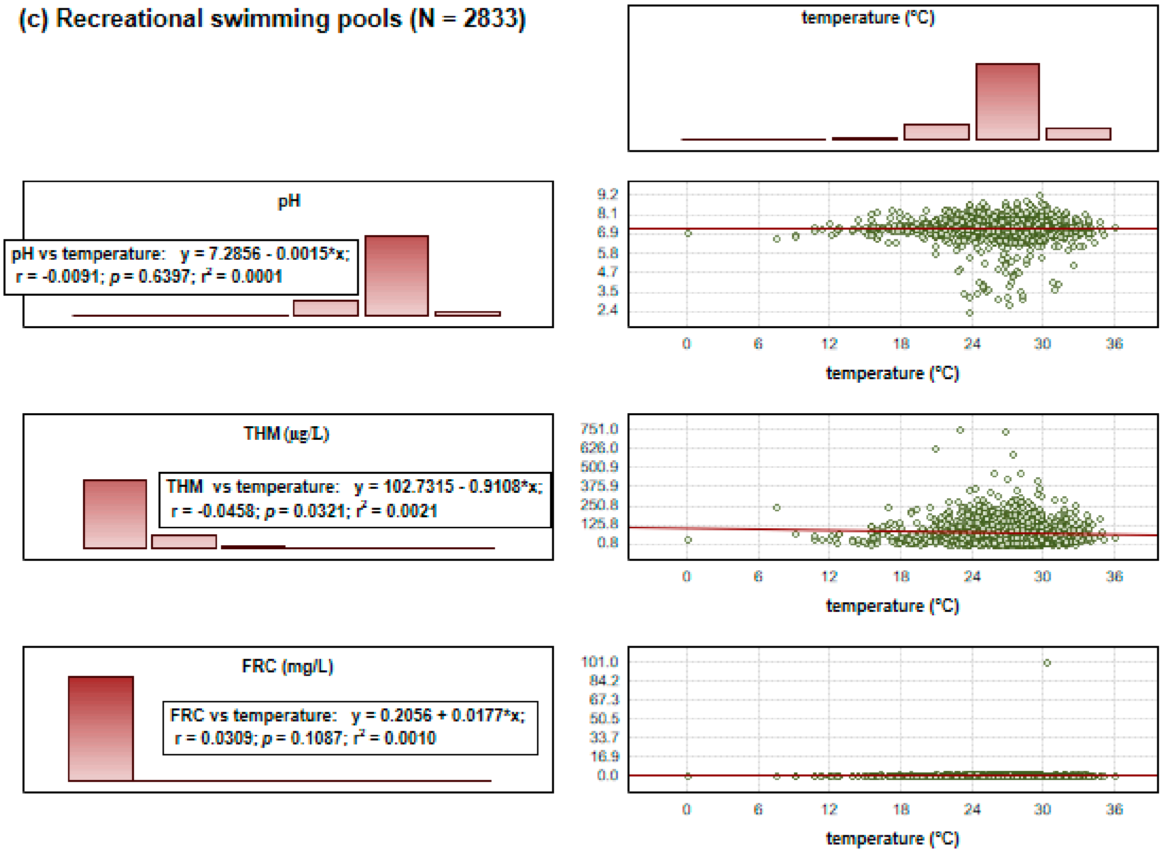

(d) Hot tubs (N = 966)

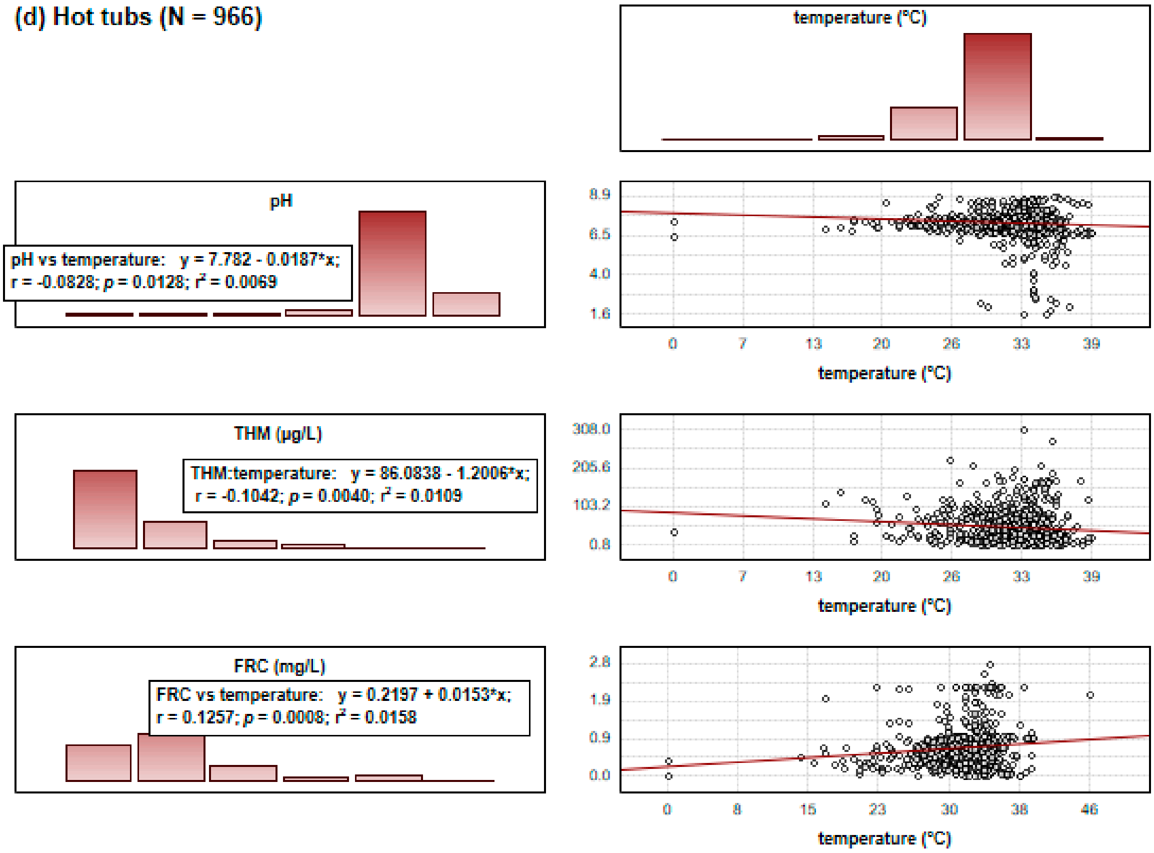

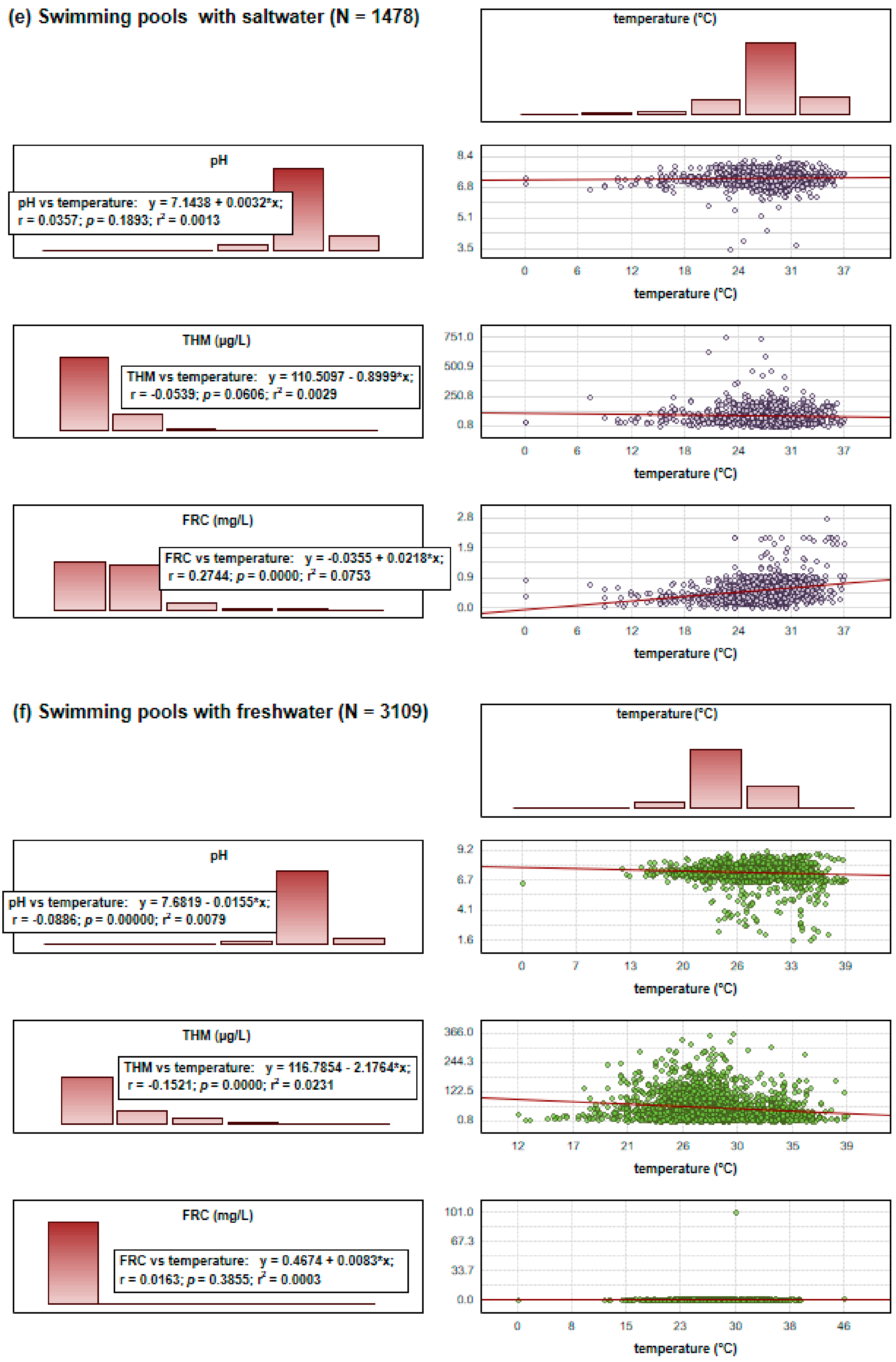

Figure S2. Correlations between temperature and physico-chemical characteristics (pH, THM, FRC) of waters in: a) outdoor , b) indoor, c) recreational swimming pools, d) hot tubs, e) swimming pools with salt water, and f) swimming pools with fresh water.

Table S1. Eigenvector values and variable contributions for analyzed swimming water pools' physico-chemical parameters (temperature (°C), pH, trihalomethanes (THM; μg/L), free residual chlorine (FRC; mg/L)), and bacteria: *Legionella pneumophila* (CFU/L), depending on whether it was an indoor or outdoor swimming pool and the type of swimming pool (children´s, recreational, hot tub, or sports/Olympic), as determined by principal component analysis (PCA) with first three main components (PC 1, PC 2 and PC 3). ($N_{pool\ data}$ = 4587; $N_{outdoor}$ = 2233; N_{indoor} = 2354).

Variable	Eigenvector spreadsheet		
	PC 1	PC 2	PC 3
temperature	0.43	−0.43	0.29
pH	−0.45	0.19	−0.60
THM	0.53	0.30	−0.41
FRC	0.57	0.21	−0.31
L. pneumophila	0.01	−0.80	−0.53
	Variable contribution		
	PC 1	PC 2	PC 3
temperature	0.19	0.19	0.08
pH	0.20	0.04	0.37
THM	0.29	0.09	0.17
FRC	0.33	0.04	0.10
L. pneumophila	0.01	0.64	0.28

Table S2. Eigenvector values and variable contributions for analyzed swimming water pools' physico-chemical parameters (temperature (°C), pH, trihalomethanes (THM; μg/L), free residual chlorine (FRC; mg/L)), and bacteria: *Legionella pneumophila* (CFU/L), depending on water in swimming pool (saltwater or freshwater) and type of swimming pool (children´s, recreational, hot tub, or sports/Olympic), as determined by principal component analysis (PCA) with first three main components (PC 1, PC 2 and PC 3). ($N_{pool\ data}$ = 4587; $N_{saltwater}$ = 1478; N_{indoor} = 3109).

Variable	Eigenvector spreadsheet		
	PC 1	PC 2	PC 3
temperature	0.45	−0.62	−0.02
pH	−0.32	−0.55	−0.26
THM	0.48	0.49	−0.02
FRC	0.68	−0.19	−0.08
L. pneumophila	−0.01	−0.17	0.96

	Variable contribution		
	PC 1	PC 2	PC 3
temperature	0.20	0.39	0.01
pH	0.10	0.31	0.07
THM	0.23	0.24	0.01
FRC	0.47	0.04	0.01
L. pneumophila	0.01	0.03	0.93
